# Supplementary material for: HIVprotI: an integrated web based platform for prediction and design of HIV proteins inhibitors
Source: J Cheminform. 2018 Mar 9;10:12. doi: 10.1186/s13321-018-0266-y (PMC5845081; doi:10.1186/s13321-018-0266-y)
Supplement: Supplementary file 2 — Additional file 2. Source code of HIVProtI web server. [file 13321_2018_266_MOESM2_ESM.zip › HIVprotI_Source-code/hivprotI/weka-3-6-6/doc/weka/associations/AprioriItemSet.html]

AprioriItemSet


---


|  |  |  |
| --- | --- | --- |
| **Overview** \| **Package** \| **Class** \| **Tree** \| **Deprecated** \| **Index** \| **Help** \| **Weka's home** | |  |
| **PREV CLASS**   **NEXT CLASS** | **FRAMES**    **NO FRAMES**     **All Classes** |  |
| SUMMARY: NESTED \| FIELD \| CONSTR \| METHOD | DETAIL: FIELD \| CONSTR \| METHOD |  |


---


## weka.associations Class AprioriItemSet

```
java.lang.Object
  weka.associations.ItemSet
      weka.associations.AprioriItemSet
```

**All Implemented Interfaces:**: java.io.Serializable, RevisionHandler

---

``` public class AprioriItemSet extends ItemSet implements java.io.Serializable, RevisionHandler ```

Class for storing a set of items. Item sets are stored in a lexicographic
order, which is determined by the header information of the set of instances
used for generating the set of items. All methods in this class assume that
item sets are stored in lexicographic order.
The class provides methods that are used in the Apriori algorithm to construct
association rules.

**Version:**
:   $Revision: 1.6 $

**Author:**
:   Eibe Frank (eibe@cs.waikato.ac.nz), Stefan Mutter (mutter@cs.waikato.ac.nz)

**See Also:**: Serialized Form

---

**Constructor Summary**

`AprioriItemSet(int totalTrans)`
  
          Constructor


| **Method Summary** | |
| --- | --- |
| `static double` | `confidenceForRule(AprioriItemSet premise, AprioriItemSet consequence)`             Outputs the confidence for a rule. |
| `double` | `convictionForRule(AprioriItemSet premise, AprioriItemSet consequence, int premiseCount, int consequenceCount)`             Outputs the conviction for a rule. |
| `FastVector[]` | `generateRules(double minConfidence, FastVector hashtables, int numItemsInSet)`             Generates all rules for an item set. |
| `FastVector[]` | `generateRulesBruteForce(double minMetric, int metricType, FastVector hashtables, int numItemsInSet, int numTransactions, double significanceLevel)`             Generates all significant rules for an item set. |
| `java.lang.String` | `getRevision()`             Returns the revision string. |
| `double` | `leverageForRule(AprioriItemSet premise, AprioriItemSet consequence, int premiseCount, int consequenceCount)`             Outputs the leverage for a rule. |
| `double` | `liftForRule(AprioriItemSet premise, AprioriItemSet consequence, int consequenceCount)`             Outputs the lift for a rule. |
| `static FastVector` | `mergeAllItemSets(FastVector itemSets, int size, int totalTrans)`             Merges all item sets in the set of (k-1)-item sets to create the (k)-item sets and updates the counters. |
| `static FastVector` | `singletons(Instances instances)`             Converts the header info of the given set of instances into a set of item sets (singletons). |
| `AprioriItemSet` | `subtract(AprioriItemSet toSubtract)`             Subtracts an item set from another one. |
| `java.lang.String` | `toString(Instances instances)`             Returns the contents of an item set as a string. |

**Methods inherited from class weka.associations.ItemSet**

`containedBy, counter, deleteItemSets, equals, getHashtable, hashCode, itemAt, items, pruneItemSets, pruneRules, setCounter, setItem, setItemAt, support, upDateCounter, upDateCounters`

**Methods inherited from class java.lang.Object**

`getClass, notify, notifyAll, toString, wait, wait, wait`

**Constructor Detail**

### AprioriItemSet

```
public AprioriItemSet(int totalTrans)
```

:   Constructor

    **Parameters:**: `totalTrans` - the total number of transactions in the data


**Method Detail**

### confidenceForRule

```
public static double confidenceForRule(AprioriItemSet premise,
                                       AprioriItemSet consequence)
```

:   Outputs the confidence for a rule.

    :   **Parameters:**: `premise` - the premise of the rule: `consequence` - the consequence of the rule **Returns:**: the confidence on the training data

---


### liftForRule

```
public double liftForRule(AprioriItemSet premise,
                          AprioriItemSet consequence,
                          int consequenceCount)
```

:   Outputs the lift for a rule. Lift is defined as:  
    confidence / prob(consequence)

    :   **Parameters:**: `premise` - the premise of the rule: `consequence` - the consequence of the rule: `consequenceCount` - how many times the consequence occurs independent of the premise **Returns:**: the lift on the training data

---


### leverageForRule

```
public double leverageForRule(AprioriItemSet premise,
                              AprioriItemSet consequence,
                              int premiseCount,
                              int consequenceCount)
```

:   Outputs the leverage for a rule. Leverage is defined as:   
    prob(premise & consequence) - (prob(premise) \* prob(consequence))

    :   **Parameters:**: `premise` - the premise of the rule: `consequence` - the consequence of the rule: `premiseCount` - how many times the premise occurs independent of the consequent: `consequenceCount` - how many times the consequence occurs independent of the premise **Returns:**: the leverage on the training data

---


### convictionForRule

```
public double convictionForRule(AprioriItemSet premise,
                                AprioriItemSet consequence,
                                int premiseCount,
                                int consequenceCount)
```

:   Outputs the conviction for a rule. Conviction is defined as:   
    prob(premise) \* prob(!consequence) / prob(premise & !consequence)

    :   **Parameters:**: `premise` - the premise of the rule: `consequence` - the consequence of the rule: `premiseCount` - how many times the premise occurs independent of the consequent: `consequenceCount` - how many times the consequence occurs independent of the premise **Returns:**: the conviction on the training data

---


### generateRules

```
public FastVector[] generateRules(double minConfidence,
                                  FastVector hashtables,
                                  int numItemsInSet)
```

:   Generates all rules for an item set.

    :   **Parameters:**: `minConfidence` - the minimum confidence the rules have to have: `hashtables` - containing all(!) previously generated item sets: `numItemsInSet` - the size of the item set for which the rules are to be generated **Returns:**: all the rules with minimum confidence for the given item set

---


### generateRulesBruteForce

```
public final FastVector[] generateRulesBruteForce(double minMetric,
                                                  int metricType,
                                                  FastVector hashtables,
                                                  int numItemsInSet,
                                                  int numTransactions,
                                                  double significanceLevel)
                                           throws java.lang.Exception
```

:   Generates all significant rules for an item set.

    :   **Parameters:**: `minMetric` - the minimum metric (confidence, lift, leverage, improvement) the rules have to have: `metricType` - (confidence=0, lift, leverage, improvement): `hashtables` - containing all(!) previously generated item sets: `numItemsInSet` - the size of the item set for which the rules are to be generated: `numTransactions` -: `significanceLevel` - the significance level for testing the rules **Returns:**: all the rules with minimum metric for the given item set **Throws:**: `java.lang.Exception` - if something goes wrong

---


### subtract

```
public final AprioriItemSet subtract(AprioriItemSet toSubtract)
```

:   Subtracts an item set from another one.

    :   **Parameters:**: `toSubtract` - the item set to be subtracted from this one. **Returns:**: an item set that only contains items form this item sets that are not contained by toSubtract

---


### toString

```
public final java.lang.String toString(Instances instances)
```

:   Returns the contents of an item set as a string.

    :   **Overrides:**: `toString` in class `ItemSet`
    :   **Parameters:**: `instances` - contains the relevant header information **Returns:**: string describing the item set

---


### singletons

```
public static FastVector singletons(Instances instances)
                             throws java.lang.Exception
```

:   Converts the header info of the given set of instances into a set
    of item sets (singletons). The ordering of values in the header file
    determines the lexicographic order.

    :   **Parameters:**: `instances` - the set of instances whose header info is to be used **Returns:**: a set of item sets, each containing a single item **Throws:**: `java.lang.Exception` - if singletons can't be generated successfully

---


### mergeAllItemSets

```
public static FastVector mergeAllItemSets(FastVector itemSets,
                                          int size,
                                          int totalTrans)
```

:   Merges all item sets in the set of (k-1)-item sets
    to create the (k)-item sets and updates the counters.

    :   **Parameters:**: `itemSets` - the set of (k-1)-item sets: `size` - the value of (k-1): `totalTrans` - the total number of transactions in the data **Returns:**: the generated (k)-item sets

---


### getRevision

```
public java.lang.String getRevision()
```

:   Returns the revision string.

    :   **Specified by:**: `getRevision` in interface `RevisionHandler` **Overrides:**: `getRevision` in class `ItemSet`
    :   **Returns:**: the revision


---


|  |  |  |
| --- | --- | --- |
| **Overview** \| **Package** \| **Class** \| **Tree** \| **Deprecated** \| **Index** \| **Help** \| **Weka's home** | |  |
| **PREV CLASS**   **NEXT CLASS** | **FRAMES**    **NO FRAMES**     **All Classes** |  |
| SUMMARY: NESTED \| FIELD \| CONSTR \| METHOD | DETAIL: FIELD \| CONSTR \| METHOD |  |


---
